# Severe Housing Deprivation in the European Union: a Joint Analysis of Measurement and Theory

**DOI:** 10.1007/s11205-022-02987-6

**Published:** 2022-08-24

**Authors:** Rod Hick, Marco Pomati, Mark Stephens

**Affiliations:** 1 School of Social Sciences, Cardiff University, Cardiff, UK; 2 School of Social and Political Sciences, University of Glasgow, Glasgow, UK

**Keywords:** Housing deprivation, Poverty, Europe, Measurement, Theory, Overcrowding

## Abstract

Concerns about the quality of housing feature prominently in academic and policy discussion on housing, yet there is little agreement about how housing deprivation should be measured or monitored. In empirical studies, measures of housing deprivation are typically examined for one of two purposes—either to compare incidences of housing quality problems for different groups, which typically leads to an examination of performance of different measures of housing deprivation, or as dependent variables to examine competing theories about what explains cross-national variation in such problems, which typically ignores these measurement considerations. Our paper seeks to analyse measurement and theory jointly, focussing in particular on the EU’s severe housing deprivation measure, and its subcomponents—overcrowding and housing conditions problems. In descriptive analysis, we show that the two components of the severe housing deprivation measure are weakly related and pattern differently across nations and that the aggregation rule of the main measure has a substantial influence on observed incidences of this problem. We subsequently construct multi-level regression-based models and demonstrate that the two components have quite different determinants. Our paper has implications for the measurement of severe housing deprivation in Europe, for theories that seek to account for differences in housing outcomes, and for policy that seeks to tackle housing deprivation problems.

## Introduction

Concerns about the quality of housing feature prominently in academic and policy discussion on housing, yet there is little agreement about how housing deprivation should be measured or monitored. One immediate challenge is that the monitoring of population living standards is typically based on the analysis of statistics derived from surveys of private households, yet the sampling frames for such studies exclude many of the very people who might be said to be suffering the most extreme forms of unmet shelter need, such as those experiencing rooflessness or living in various forms of supported housing.

Even for the population living in private households, there is limited agreement that housing deprivation constitutes one clearly-defined problem or even a small number of related-but-distinct problems. In their examination of housing deprivation amongst older people in Ireland, Nolan & Winston, 2011: 37) suggest that the term ‘“housing problems” may be used to cover a variety of rather different circumstances’, while a recent report by the OECD, 2021: 1) claims that ‘No agreed definition of (severe) housing deprivation exists across countries’.

In policy terms, one prominent measure of housing inadequacy is the EU’s official measure of severe housing deprivation. The EU’s statistical agency Eurostat monitors the prevalence of a range of housing quality problems—namely, the percentage of individuals living in households with a leaking roof, with no bath and shower and no indoor toilet, or in a dwelling considered too dark. These are labelled *housing deprivations* (or, what we label in this paper the deprivation of housing ‘conditions’). The EU also reports a measure of *severe housing deprivation*, which reflects the circumstance where households experience at least one of these deprivations in housing conditions *and* also experiences overcrowding.

At the European level, there has been a desire—but also a reluctance—to elevate a measure of housing problems in the core list of ‘primary’ social indicators because of a lack of agreement about the form such an indicator should take (EU Social Protection Committee and Indicators Sub-group, 2015). Secondary indicators for both housing affordability and a measure of housing deprivation (but not *severe* housing deprivation, incorporating the experience of overcrowding, too) exist however and, more recently, housing conditions and overcrowding have been included separately as headline indicators to monitor progress as part of the EU Roma strategic framework for Equality, Inclusion and Participation (European Commission, 2020b: 3). Nonetheless, the EU’s severe housing deprivation indicator has itself not been endorsed in these monitoring and policy frameworks. This ambivalence reflects concerns over the statistical performance of the measure, but also a more general lack of agreement about the essence of housing quality problems. This lack of agreement in relation to conceptualisation and measurement is problematic given the desire for such data to inform political and public deliberation on housing standards (see also Stephens & Hick, forthcoming).

In academic and policy literature, estimates of (severe) housing deprivation are typically examined for one of two main purposes. The first is to understand the incidence of housing quality problems for different households, socio-demographic groups or countries (e.g. Ulman & Ćweik, 2021). This branch of literature often includes discussion of how performance differs by indicator of housing deprivation, which typically leads to a focus on how housing deprivation is measured. The second is to use measures of (severe) housing deprivation to investigate theoretical claims about the determinants of housing outcomes more generally—for example, examining whether the structure of housing systems or economic factors such as per capita income best explains variations in housing outcomes across Europe (e.g. Borg, 2015). Importantly, these two purposes have seldom been combined in the one study which, in relation to the second purpose, has meant that theoretical discussion about the causes of housing outcomes is not always as attentive to concerns about the conceptualisation and measurement of the dependent variable as it might be.

In this paper, we examine the nature, components and determinants of severe housing deprivation in Europe. In doing so, we seek to significantly extend the evidence base: by both examining the nature of the severe housing deprivation measure and its components, thus better understanding the nature of these indicators, as well as examining their determinants—by analysing how they perform in theoretically-significant ways. We have argued elsewhere that the ‘dependent variable problem’, on which much has been written in the comparative welfare state literature, has not received the same degree of attention in housing studies (Hick & Stephens, forthcoming). In the welfare state literature, the dependent variable problem has come to refer to questions about conceptualisation and measurement (Green-Pedersen, 2007) and centres on the issue that alternative operationalisations of the welfare state lead to substantially different perspectives about the *extent* of change. This frustrates theorising since there is not agreement about the extent of variation and/or change to be explained. Our aim is to interrogate severe housing deprivation and its components and, in so doing, contribute to theoretical debates about the determinants of housing standards.

## The Meaning and Measurement of Housing Deprivation

The concept of housing deprivation is used in the literature to refer to a number of unsatisfactory housing situations. Indeed, some analysts employ the concept of (severe) housing deprivation to refer to situations of homelessness—circumstances where a person lacks access to minimally adequate housing (Busch-Geertsema et al*.*, 2016: 125). This might include, but is not limited to, circumstances of street homelessness or living in temporary shelters (Amore, 2016) or living with friends ‘for lack of a home of one’s own’ (Brousse, 2004: 6). The FEANTSA European Typology of Homelessness and Housing Exclusion (ETHOS) framework contains four categories—relating to being roofless, houseless, living in insecure and in inadequate housing (FEANTSA, n.d.). Thus, this framework spans homelessness and housing deprivation, as we consider it here. The EU measure of severe housing deprivation, which is our primary reference point here, differs from these concepts in that having some form of housing is a precondition for experiencing housing deprivation.

The measurement and monitoring of housing deprivation is frustrated by four issues. First, the most egregious issue is, as we have noted above, that many of the most severely deprived in terms of housing—those who experience street homelessness, but also those living in sheltered accommodation, caravans and so forth, do not meet the definition of ‘private households’ and consequently are excluded from almost all household surveys. This reliance on household surveys presents a significant problem since, while providing rich information, they preclude a focus on the most extreme forms of housing deprivation that we might wish to consider.

There are then, and second, differences in terms of the dimensions of housing deprivation covered in different studies. In their review of the literature on housing deprivation, focussing specifically on household surveys, Eurofound (2016: 13, 27) find that most studies examine three domains—basic facilities, structural problems and overcrowding—though some include the first two but not overcrowding, and others incorporate wider measures of neighbourhood deprivation. But some approaches are broader still. The reliance on lockdown measures during the COVID-19 pandemic has led to a growth in interest in housing circumstances, and Ayala et al. (2021) present an analysis of a broad measure of housing deprivation that includes, inter alia, whether a dwelling is located in a densely-populated area and whether it has internet access.

Third, there are perceived issues relating to some of the specific indicators used in the Eurostat measure and key studies. Winston and Kennedy (2019) note that the prevalence of some subcomponents—namely, indoor toilet, and bath and shower are very low and thus breach Eurostat guidelines. Lelkes with Zólyomi (2010: 9) show that “objective” measurements of overcrowding, which impose a common threshold on all nations, produce very different country orderings than those based on self-assessment. This has led to suggestions that the objective measure lacks face validity and inspired Sunega and Lux (2016) to try to construct alternative objective overcrowding measures, based on nationally-variant thresholds, that are in closer alignment with subjective country-rankings. This approach foregrounds the important question of whether benchmarks should be European or national; one limitation is that, in effect, it treats the subjective rankings as valid, to which the objective measure should be adjusted accordingly. The field of poverty analysis, too, has wrestled with the question of whether poverty thresholds should be national, pan-European, or some combination of the two (e.g. Fahey, 2007).

The fourth and final consideration relates to differences in the extent to which dimensions are analysed separately or as a composite measure and discussion in relation to this consists of both principled and empirically-determined arguments. In relation to the former, Clair et al. (2019) favour a broader, multidimensional measure of housing ‘precariousness’, a composite measure including the dimensions of security, affordability, quality and access to services. But Palvarini and Pavloni (2010) find that the relationship between distinct housing problems is weak and Eurofound (2016: 18) insist that affordability considerations are fundamentally distinct from concerns about the adequacy of the dwelling and should therefore be considered separately. Guio & Engsted Maquet, 2007: 201) observe that, despite overcrowding being weakly related to other housing and material deprivation items, which might merit exclusion on empirical grounds, ‘most of the Member States consider this information as a crucial one’.

Nolan and Winston (2011) ask, relatedly, whether the four different measures of housing problems they identify (poor housing quality, lack of household consumer durables, housing cost, and neighbourhood problems) can be considered to reflect a measure of ‘multiple deprivation’. They show that the correlation between these four measures is low and that they capture different groups. They conclude:

‘adding together the different dimensions of housing-related deprivation masks rather than reveals the underlying social structuring of housing-related deprivation, because these dimensions are only loosely related to each other. This brings out the importance of distinguishing and studying these dimensions separately and *framing appropriate policy responses in that light*’ (Nolan and Winston, 2011: 382, emphasis is ours).

All of this matters because there is a tendency within the literature to seek to explore either the incidence of housing problems amongst certain groups or to use these measures as dependent variables to evaluate competing theories about what drives differences in housing outcomes across nations. But if there is not agreement about the outcome variable of interest, and if competing alternatives perform quite differently in empirical terms, then these competing operationalisations may provide a contradictory guide to performance and may lend support to quite different theories explaining housing outcomes. It is to these competing explanations that we now turn.

## Explaining Differences in Housing Standards

In addition to monitoring differences in housing standards, one way in which housing deprivation data is often used is as a dependent variable in studies seeking to explore the determinants of such standards. In the literature on housing deprivation, there are two primary explanations for variations in housing quality across countries. The first is that a country’s level of economic development, as captured by GDP per capita, is the key determinant of housing conditions (e.g. Mandic & Cirman, 2012). The chief alternative hypothesis is that housing systems (that is, country-level housing arrangements) help to protect households against, or make them vulnerable to, the experience of housing deprivation (e.g. Norris & Sheils, 2007).

In emphasising the wealth explanation Mandic and Cirman (2012) arrive at this conclusion based on analysis of data from the European Quality of Life Survey for 26 European countries. Their analysis of housing conditions is based on a subjective measure where respondents have reported problems in relation to a shortage of space (overcrowding); the presence of rot; damp; lacking an indoor toilet; and negative perception of the safety of the neighbourhood. Of the five explanatory variables they consider, they find that GDP per capita has the strongest association with housing conditions problems.

Our paper is drawn from a wider project examining the relationship between housing and poverty in Europe, so we are also interested in economic disadvantage in a distribution-sensitive manner—namely, via rates of poverty and material deprivation. In this vein, we examine the at risk of poverty rate, a relative measure set at 60 per cent of national equivalised median income and a measure of material deprivation. The latter asks households about a series of nine necessities, such as whether they are able to face unexpected expenses, whether they own a washing machine or go on an annual holiday, building on the work of Peter Townsend (1979), who pioneered their usage in the field of poverty analysis (see also Ivaldi & Ciacci, 2021). Where households do not possess these items or do not participate in these activities, they are asked whether this is because of a lack of resources. Households are given a score based on the number of items they go without because of a lack of resources and our analysis is based on the average of these scores in each country (see also Nolan & Whelan, 2011, for analysis of these two poverty measures). When compared with the at-risk-of-poverty rate, this measure is much more concentrated on the nations of Central and Eastern Europe, which have lower real living standards (Hick et al., 2022).

Arguments in favour of housing systems often build on the work of Jim Kemeny (1992, 1995). Kemeny examined differences in housing systems in eight countries, arguing that the central differences between housing systems originated from the nature of their rental markets—between what he labelled dual and unitary rental systems. In the unitary system, the state’s provision of extensive cost rental housing enables this sector to compete with the private rental market. This competition helps to drive down costs and to set a floor on housing standards, leading, ultimately, to a unitary rental system. By contrast, in the dualist system, the state’s desire to incentivise the profit-orientation of the rental market leads it to adopt a minimalist cost rental sector, which, since the private market is unlikely to be able to meet the housing needs of ordinary households satisfactorily, shapes preferences in favour of owner-occupation. Importantly, Kemeny is clear that these different types of system will have predictable consequences in terms of housing outcomes: unitary systems are understood as having the potential to lower rents charged in the market rental sector and to set a floor on housing standards (e.g. Kemeny, 1995: 132, 18), in contrast with dualist systems.

Empirical work in this tradition, then, tends to measure differences in housing systems based on some measure of tenure balances. Borg (2015) analyses data for the EU severe housing deprivation measure for 25 European countries in 2007 and finds that the size of the market rental sector (which she labels the integrated rental sector) ‘explains a great proportion of the variations in the prevalence of housing deprivation’ (2015: 91), with no association observed in relation to GDP per capita. Borg and Guio (2021) expand the analysis of tenure balance measures and qualify this finding by suggesting that the proportion of outright ownership is what matters for explaining severe housing deprivation and, controlling for this, that the size of the rental sector ceases to matter. They find that levels of wealth are, separately, related to severe housing deprivation, but do not jointly test wealth and tenure balance explanations. Norris and Shiels (2007) examine country-level policy data drawn from multiple sources for 25 European nations. They find that a measure of housing conditions consisting of housing quality, accessibility and affordability is only weakly related to GDP per capita across the EU and conclude that ‘historical differences in the availability of public and private finance for housing have contributed to poorer housing standards in the southern than in the northern states of the EU-15’. Mandic and Cirman (2012: 789) observe a statistically significant relationship between the country-level homeownership rate and housing conditions problems but, as noted above, find this to be of secondary importance to the dominating consideration of average wealth.

In recent years, accounts of housing system change have turned towards the process of financialisation which, it is claimed, increasingly explains the way that housing systems are changing (e.g. Stephens, 2020). Accounts of financialisation take a variety of forms, but typically centre on how the greater availability of mortgage finance, ultra-low interest rates and higher rates of permitted leverage combine to inflate asset prices, limiting access in particular for those on low incomes and for the young (where familial financial support is absent) (see also Hick & Stephens, forthcoming, for a discussion). This explanatory process relates more directly to the *affordability* of housing than to its quality, but quality and cost considerations are not unrelated—a family might, for instance, accept living in an overcrowded or otherwise low-quality dwelling because adequate housing costs too much. Two prominent empirical proxies of housing market financialisation are Total Residential Loans as a proportion of GDP (Schwartz & Seabrooke, 2008) and the proportion of households who are mortgaged homeowners (Hick & Stephens, forthcoming), and we explore these here in relation to severe housing deprivation. In examining country-level differences in severe housing deprivation we draw both on housing-specific as well as economic explanations.

The three questions we look to address in this paper are:

1. What is the relationship between the component items of the severe housing deprivation measure? Does this vary between countries?
2. What socio-economic groups are at risk of severe housing deprivation and its components? In particular, how do ownership and dwelling characteristics relate to these problems?
3. What countries have higher rates of severe housing deprivation and its subcomponents? To what extent are elevated rates explained by average wealth, poverty, and the financialisation of housing?

## Data and Method

The analysis presented in this paper is based on the EU Statistics on Income and Living Conditions survey (EU-SILC). EU-SILC is the primary European survey for examining household living standards and it contains a number of relevant housing-related variables. At the same time, only private households—that is, members who ‘share expenditures’—are included, so members of collective households, such as those living in student dorms, residential homes, and people living in ‘institutions for asylum seekers and migrant workers’ are excluded (Eurostat, n.d.). The unit of analysis in this paper is the household, as defined in EU-SILC. Our analysis is based on data from 2016, the most recent year that micro-data on severe housing deprivation that includes the UK is available. In this analysis we pay particular attention to the EU Severe Housing Deprivation measure and its components. Our initial plan was to analyse the incidence of housing deprivation through time. However, based on extensive initial descriptive work we decided that this was not possible because (i) there is some item missingness in some countries in some years, but also (ii) there appeared to be implausibly large year-on-year change in some items, which seemed to us to more likely capture error rather than true variation. This relates to the less-than-ideal measurement of housing variables in SILC, which we have argued elsewhere are in need of greater attention by Eurostat (Hick et al., 2022).

We have sought to present analysis of the “old” EU-28 (that is, including the UK). Our final analysis is based on data from 27 countries—Germany is excluded as it did not collect data for the bath/shower questions in 2016. For some of the descriptive analysis we classify countries into welfare regimes as a means of presenting the data more clearly. These welfare regimes are themselves correlated with wealth so presenting descriptive information in this way allows us to observe the extent to which housing deprivation is concentrated amongst poorer Member States in Central and Eastern Europe, or is more widely experienced.

The EU’s official measure of severe housing deprivation, as noted, comprises five component indicators. These are defined as follows.

- *Overcrowding.* The overcrowding measure seeks to capture inadequate space relative to a household’s space needs. EUROSTAT defines a dwelling as overcrowded if it lacks a room for: each household and in addition to this a room for: each couple in the household; each single person aged 18 or more; each pair of single people of the same sex between 12 and 17 years of age; each single person between 12 and 17 years of age and not included in the previous category; each pair of children under 12 years of age.
- *Darkness.* The darkness indicator identifies households where the household respondent reports that their dwelling is too dark, meaning that there is not enough daylight coming through the windows during the day.
- *Leak.* The leak indicator identifies households where the household respondent reports that there are any of the following issues in the dwelling: a leaking roof and/or damp ceilings, dampness in the walls, floors or foundation and/or rot in window frames and doors. While it is common to use the term ‘leak’ to describe this indicator, it is important to be mindful that its full definition is somewhat broader.
- *Indoor bath/shower and indoor flushing toilet for the sole use of their household.* Of particular note here is that the bath & shower and toilet indicators are measured separately but are counted as one item for the purpose of the severe housing deprivation measure. A household is classified as deprived under this indicator only if they lack both items (and is therefore an intersection approach). But outside of the official severe housing deprivation measure, these items are treated as being distinct (e.g. Rybkowska and Schneider, 2011), including in the EU’s own measurement of housing deprivation incorporated as a secondary indicator in its social indicators monitoring framework.

These indicators of housing deprivation are reasonably intuitive but overcrowding requires some explanation. It is based on the relationship between the number of rooms and number, sex and relationships of household members. The former counts only rooms greater than four square metres in size and which are available for living—so, bedrooms and living/dining rooms are counted; halls, landings, utility rooms, bathrooms and balconies are not. Kitchens are not counted if they are used solely for cooking. Multiple household dwellings are assumed to share rooms equally, so for example available rooms are divided by two if there are two households in a dwelling. Of importance is the method for determining the number of “required” rooms: each household, before we know anything of its membership, is assumed to require one room; once we start to add people, they immediately require a second room. The consequence of this definition is that *all* one-room dwellings are over-crowded; two-room dwellings are over-crowded if there are children in the dwelling as well as adults, or if there are two adults, but they are not a couple.

These indicators have been statistically validated by us using the subjective satisfaction with housing measure contained in the 2012 ad hoc module. While objective and subjective measures of many social problems are far from perfectly correlated (e.g. Ivaldi et al*.*, 2018), we expect that, within countries, respondents experiencing housing deprivation, objectively-speaking, will report higher levels of dissatisfaction with housing. Subjective indicators also have precedent in terms of being used as tests of validity for measures of material deprivation (see Guio et al., 2016). The findings (which we do not show here) demonstrate that being deprived on any of the indicators results in substantially higher incidence of dissatisfaction with housing.

Our analytic approach, which is novel in terms of considering both measurement and theory, proceeds in stages. In Part 1 we provide headline estimates of the component indicators of the severe housing deprivation measure and analysis of their inter-relations and explore how the incidence of housing deprivation varies for two key variables: welfare regime clusters and income quintiles. In Part 2, we explore the incidence of housing deprivation across a wider range of socio-economic characteristics. Part 3 presents the results from a series of multi-level models in which we control for the aforementioned characteristics, but also estimate country-level random effects. The advantage of the random effects model is that it allows country-level co-variates to be considered, which enables us to model the effects of the rate of mortgaged homeownership, poverty rates and GDP per capita on housing deprivation, while also controlling for compositional differences.

In terms of our modelling strategy, we use multilevel logit models to estimate the probability of overcrowding, conditions deprivation and combined deprivation (i.e. the EU severe housing deprivation indicator). Specifically, we estimate a two-level model with households (level-1 units) nested within countries (level-2 units). All multilevel analyses were fitted in R using rescaled survey weights that sum to the cluster sample size (Asparouhov, 2006). To explore the importance of country- and individual-level variation we compare models using intraclass correlation (ICC), median odds ratios (MOR) and Nakagawa et al.’s R-Squared (2017). The latter distinguishes between marginal and conditional R-squared, considering variance explained by the fixed effects and the variance explained by both fixed and random effects.

## Analysis

### Part 1—Headline estimates of housing deprivation problems

**Fig. 1:**
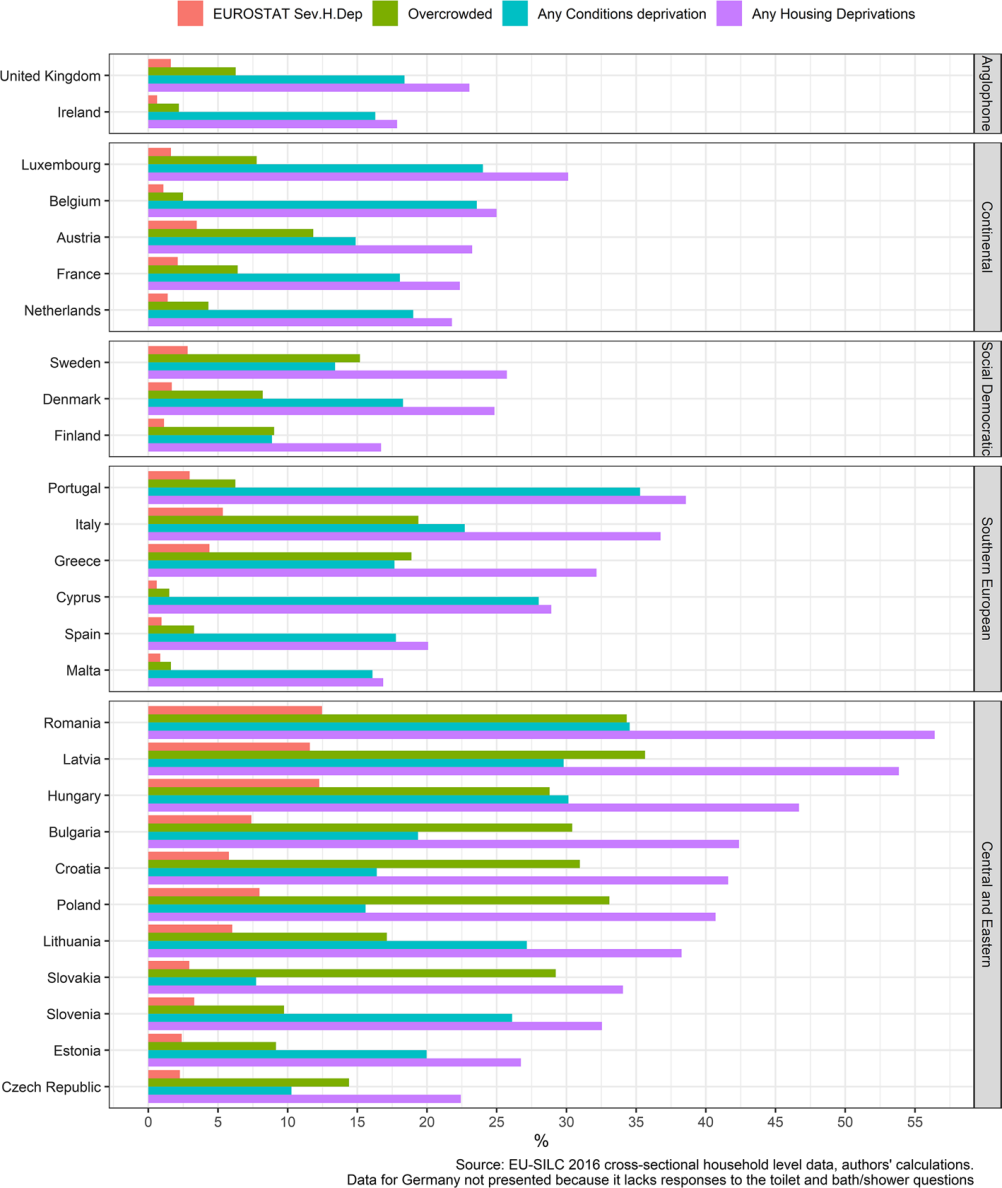
Housing deprivation—official measure and alternative indicators

Our analysis begins by examining the incidence of the official severe housing deprivation measure by country (represented by the red bars in Fig. 1), where the countries have been clustered into welfare regimes. We immediately see that the incidence of severe housing deprivation is concentrated primarily in some of the Central and Eastern European nations and is very low in the Anglophone, Continental and Social Democratic worlds as well as in parts of Southern Europe. Indeed, in a full half of our sample (14/27 countries), the incidence of severe housing deprivation falls below 3% of households. Taken at face value, this seems to suggest that housing quality problems are marginal in much of Europe. It may, alternatively, be indicative of a measurement construct that is too difficult (in statistical terms) or is poorly specified, for the richer nations of Europe in particular.

To consider this further, we have also plotted the incidence of the two components of the severe housing deprivation measure—overcrowding and conditions deprivation—as well as a summary measure reflecting the incidence of *any* of these housing deprivations (the purple bar). These paint very different patterns of incidence of the elements of housing deprivation. If we focus on the experience of *any* of the housing deprivation items (the purple bars), we see that rates exceed 50 per cent in Romania and Latvia, are above 30 per cent in all of the Central and Eastern European nations (bar the Czech Republic and Estonia) and in three nations in Southern Europe (Portugal, Italy and Greece). The incidence of housing deprivation on this measure is not negligible even in the richer parts of Europe, with rates of around 15–25 per cent in many countries in the Social Democratic, Continental and Anglophone worlds. We see from this that the conclusion that housing quality problems are marginal in much of Europe is very much a result of the way that the severe housing deprivation measure is constructed.

Considering the two elements of the severe housing deprivation measure separately, we observe that overcrowding is considerably lower than conditions deprivation in the Anglophone countries and most nations in the Continental and Social Democratic worlds. The converse is observed in many countries in Central and Eastern Europe, where overcrowding is more common than the deprivation of conditions. This serves to demonstrate that the two elements of the severe housing deprivation measure are patterned quite differently across countries. There are also country-differences that are harder to explain by reference to these country clusters alone. For example, overcrowding is considerably lower in Malta, Spain and Cyprus than it is in Italy and Greece, and is lower than might be expected in Ireland and the UK. Deprivation in conditions is, in contrast with overcrowding, much more equally distributed across Europe. This differential patterning at the country level suggests that these two problems are driven by different causes.

The patterns discussed above are suggestive of the fact that the components of housing deprivation are not strongly correlated. We test this formally in Table 1 below, which consists of a tetrachoric correlation matrix—the appropriate correlation matrix for binary data—and find that the correlation between the items is, with exception of leak and dark, weak, and especially so between leak and dark and overcrowding. We have explored these relationships for each of the countries considered here, examining whether this correlation is greater in some parts of Europe than others (analysis which is not shown here). We find that correlations between these items in the Central and Eastern European nations, and especially between leak, dark and bath/toilet are slightly higher, but they remain low. For all countries, the correlation between overcrowding and leak and dark is below 0.3, with the exception of the correlation between overcrowding and the darkness item in Austria (0.3), Belgium (0.3) and Malta (0.4). This matters because a weak relationship between these indicators could be an artefact of the low rates of overcrowding in some nations. We find that this is not the case. The weak relationship between these indicators is not being driven by the low incidence of overcrowding in richer countries but constitutes a broader problem that is observed in all nations.

**Table 1:** Tetrachoric correlation of items contained in SHD measure

Item	Overcrowded	Toilet + Bath/Shower	Dark	Leak
Overcrowded	1.00	0.34	0.18	0.14
Toilet + Bath/Shower	0.34	1.00	0.21	0.29
Dark	0.18	0.21	1.00	0.47
Leak	0.14	0.29	0.47	1.00

Correlations calculated using household weight

**Fig. 2:**
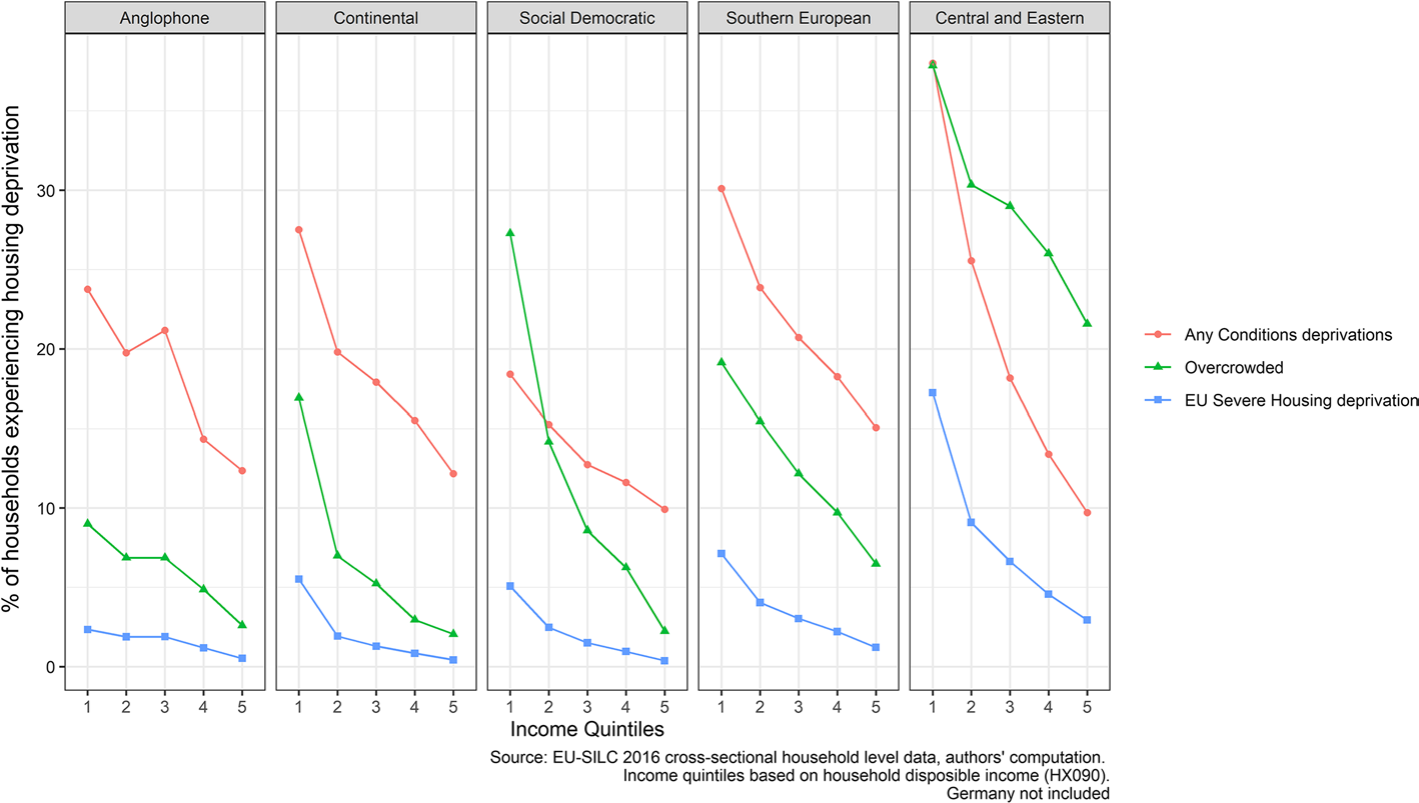
Incidence of housing derivation elements and income quintile by welfare regime

In Fig. 2 below, we examine the relationship between deprivation in housing conditions, overcrowding and severe housing deprivation by welfare regime and by income quintile. The measures capture two important differences. As we have noted above, the welfare regimes capture countries at quite different levels of wealth, especially between Central and Eastern Europe and the rest. Income quintiles are a proxy for relative deprivation, capturing households’ position on the within-country income distribution. It has previously been shown that the items that make up housing deprivation are differentially related to poverty (e.g. Eurofound, 2016: 24). We go further by exploring how this varies by different welfare regimes in Europe.

We find a linear negative association between household income and the probability of experiencing any conditions deprivation, overcrowding and severe housing deprivation, as expected. However, these relationships differ across Europe. Lacking one or more of the housing conditions is most clearly associated with income in Central and Eastern Europe and is most weakly related to income in Social Democratic nations. Patterns for the Anglophone, Continental and Southern European worlds are more similar. One striking finding is that even for households in the richest income quintile, rates of conditions deprivation do not fall below about 10 per cent in any of the worlds of welfare.

The pattern of incidence of overcrowding is different. While this is also related to one’s income position, the between-regime differences are starker, especially in relation to poorer Member States. The incidence of overcrowding in the richest quintile in Central and Eastern Europe is greater than in the poorest quintile in any of the other worlds, with the exception of the Social Democratic world (which we consider further below). Conversely, especially in the Anglophone world, overcrowding rates are only modestly related to income, never rising above 10 per cent, even for households in the lowest income quintile.

Two final points are worth noting, relating to measurement and policy respectively. First, because the severe housing deprivation measure requires overcrowding *and* lacking at least one of the housing conditions items, its incidence is low outside CEE, even for those on low incomes. The very low rates of overcrowding in the Anglophone world result in very low severe housing deprivation rates in this part of Europe. This analysis serves to show, again, that the indicators of housing deprivation perform very differently and that care is needed with the severe housing deprivation construct. Second, if “best performance” can be taken as a guide of the feasibility of improvement, then it appears easier to reduce overcrowding than deprivation of conditions to very low levels.

### Part 2: Socio-economic predictors and association with forms of housing deprivation

In this section we explore the relationship between a series of socio-economic and housing-specific predictors and the three forms of housing deprivation considered to this point: conditions deprivation (i.e. the experience of at least one of the forms of housing deprivation specified by EUROSTAT), overcrowding, and the severe housing deprivation measure, which captures the intersection between these two measures. These are shown in Table 2. This analysis looks at the incidence of these deprivations and a variety of characteristics for the full European sample. Country differences are not considered in this section but are introduced in Part 3 of the analysis.

**Table 2:** Incidence of housing quality problems by socio-economic group

Variable	Values	% Conditions deprived	% Overcrowded	% Combined (EUROSTAT)
Tenure	Outright owner	18	13	3
	Owner paying mortgage	16	6	1
	Tenant/subtenant paying rent at prevailing or market rate	26	20	6
	Accommodation is rented at a reduced rate (lower price that the market price)	25	17	5
	Accommodation is provided free	27	23	8
Dwelling type	Apartment or flat in a building with < 10 dwellings	23	17	5
	Apartment or flat in a building with > = 10 dwellings	15	21	4
	Detached house	21	10	4
	Semi-detached house	21	6	2
Area density	Thinly populated area	23	13	5
	Intermediate area	18	13	3
	Densely populated area	18	15	4
	Missing	20	5	2
Noise from neighbours or from the street	No	17	13	3
	Yes	32	16	7
Pollution, grime or other environmental problems	No	18	13	3
	Yes	34	18	7
Crime violence or vandalism in the area	No	18	13	3
	Yes	32	16	7
Housing costs burden	0–25	18	12	3
	26–50	23	15	5
	51–75	24	17	5
	76–100	27	18	6
Household composition	Single parent HH	26	23	7
	Single person HH	21	9	2
	Two adults, children	20	19	5
	Two adults, no children	17	5	2
	Other HH, no children	20	20	5
	Other	22	43	13
Country of birth	Local	19	12	3
	EU	24	15	5
	Other	25	22	8
Age	18–29	22	24	7
	30–49	21	17	5
	50–64	19	10	3
	65–80	17	6	2
N. of adults working in the HH	0	20	9	3
	1	21	16	5
	2	17	14	3
	3 +	18	27	7
IncomeQuintile	1	29	21	8
	2	22	15	4
	3	19	13	3
	4	15	11	2
	5	12	8	1
Total		20	13	4

EU-SILC 2016 cross-sectional household data (27 countries), authors' calculations

For many of the characteristics the patterning of risks of overcrowding and conditions deprivation is similar. For example, households in lower income quintiles, with higher housing cost burdens, where the household head is a non-national, or living in rented accommodation have higher rates of the two component deprivations than richer households, households with lower cost burdens, headed by a national, or owners. We could add age of household head and the presence of neighbourhood problems to this list of consistent group-orderings, but it is worth highlighting the differences in estimates for these variables. For both conditions deprivation and overcrowding, households with younger heads and who experience neighbourhood problems are at greater risk than their counterparts, but age-related incidences are especially sharp in relation to overcrowding and neighbourhood problems (noise, pollution, crime) are particularly strong predictors of deprivation in housing conditions.

There are, then, some variables where the patterning changes depending on the dependent variable being examined. This is most obvious in relation to dwelling type and area. Problems with housing conditions are more prevalent than overcrowding in houses and in apartment blocks with few dwellings, whereas overcrowding is more prevalent in large apartment blocks and is much less common amongst those living in houses. Living in a thinly-populated area is associated with an elevated risk of conditions deprivation, but overcrowding is concentrated in densely-populated areas. Thus, the patterning of these problems by dwelling type and dwelling location, two particularly important variables for any study of housing, differs fundamentally by the two subcomponents of the severe housing deprivation measure.

The construction of the severe housing deprivation measure on a ‘union’ basis, requiring the joint experience of both overcrowding and conditions deprivation results in severe housing deprivation rates which are considerably lower than those for either conditions deprivation or overcrowding on their own. The proportion who are experiencing conditions deprivation who also experience severe housing deprivation rises above one-third in only two instances (for other households with children, when it rises to 60 percent, and where there are 3 or more adults working, where it is almost 40 percent). Overlaps for those experiencing overcrowding tend to be higher but rises above 40 percent in only two instances (for two of the three neighbourhood deprivations).

If we focus on the total figures, we see that 20 per cent of the households of Europe experience conditions deprivation and 13 per cent are overcrowded, but just 4 per cent fall into the severe housing deprivation category. Perhaps most worryingly, some of the variables where discrepancies are most pronounced are housing variables (dwelling type and area).

### Part 3: Modelling these relationships

In this section, we present a series of multi-level models to examine our three main dependent variables—deprivation of conditions, overcrowding and the combined measure of severe housing deprivation. Our modelling strategy, as noted above, is to present a series of multi-level logit models for each of these dependent variables, controlling for compositional differences between countries (for example, in relation to variations in household composition, which are known to differ across Europe; see Stephens et al., 2015). Our reliance on random effects models here also enables us to consider a number of country-level variables, and these allow us to test some of the findings that have gone before in the descriptive analysis and those which are of relevance in terms of some of the theoretical debate in the field.

We present results for three sets of multi-level logistic regression models for housing conditions, overcrowding and severe housing deprivation, respectively, with the output presented across five tables. Table 4 presents the micro-level (i.e. within-country) coefficients for each of the three dependent variables. Tables 5, 6 and 7 present macro-level (i.e. between-country) coefficients for conditions deprivation, overcrowding and severe housing deprivation, respectively. Table 3 presents summary indices from all these models, quantifying the level of variation in these outcomes explained by country-level differences.

**Table 3:** Key indices of model variation from Tables 4, 5 and 6

Outcome	Fit	Null	M1 (Tenure)	M2 (Tenure +)	M3 (GDP)	M4 (Res.Loans)	M5 (% Mortg owners)	M6 (%AROP)	M7 (Mat Dep)
Conditions	ICC	0.06	0.07	0.07	0.05	0.05	0.05	0.04	0.05
	MOR	1.22	1.25	1.29	1.23	1.23	1.23	1.18	1.19
	AIC	218,104	216,116	203,169	203,165	203,167	203,167	203,160	203,162
	BIC	218,125	216,178	203,509	203,516	203,528	203,528	203,522	203,524
Overcrowding	ICC	0.27	0.32	0.29	0.19	0.18	0.18	0.18	0.18
	MOR	3.24	5.09	7.82	3.68	3.55	3.62	3.64	3.62
	AIC	167,356	160,703	132,564	132,553	132,555	132,555	132,555	132,555
	BIC	167,376	160,765	132,904	132,904	132,916	132,916	132,917	132,916
Combined	ICC	0.21	0.26	0.24	0.12	0.12	0.12	0.12	0.12
	MOR	2.27	3.26	4.42	2.05	1.99	2.02	2.04	2.01
	AIC	69,385	66,823	57,731	57,714	57,715	57,716	57,716	57,716
	BIC	69,406	66,885	58,072	58,065	58,076	58,077	58,077	58,077

**Table 4:** Multi-level models estimating incidence of housing deprivation

	Conditions		Overcrowding		Combined	
	(M1)	(M2)	(M1)	(M2)	(M1)	(M2)
Intercept	− 1.14 ***	− 0.76 ***	− 1.35 ***	− 0.76 *	− 2.86 ***	− 2.28 ***
Tenure (Market tenant)	Reference	Reference	Reference	Reference	Reference	Reference
Outright owner	− 0.42 ***	− 0.61 ***	− 1.29 ***	− 1.13 ***	− 1.24 ***	− 1.26 ***
Owner paying mortgage	− 0.52 ***	− 0.39 ***	− 1.06 ***	− 0.89 ***	− 1.19 ***	− 0.94 ***
Reduced rent	0.22 ***	0.05	0.33 ***	− 0.04	0.43 ***	0.06
Free	− 0.00	− 0.33 ***	− 0.73 ***	− 0.81 ***	− 0.36 ***	− 0.64 ***
Dwelling (Apartment/flat < = 10 dwellings)		Reference		Reference		Reference
Detached house		0.25 ***		− 0.94 ***		− 0.29 ***
Semi-detached house		0.24 ***		− 0.60 ***		− 0.22 ***
Apartment/flat > = 10 dwellings		− 0.46 ***		0.27 ***		− 0.34 ***
Area (densely populated area)		Reference		Reference		Reference
Intermediate area		− 0.04 *		− 0.21 ***		− 0.28 ***
Thinly populated area		0.22 ***		− 0.17 ***		0.02
Missing		0.03		− 0.92		− 0.76
Noise reported (No)		Reference		Reference		Reference
Yes		0.55 ***		0.03		0.46 ***
Pollution reported (No)		Reference		Reference		Reference
Yes		0.53 ***		0.10 ***		0.45 ***
Crime reported (No)		Reference		Reference		Reference
Yes		0.55 ***		0.13 ***		0.58 ***
Housing cost burden (0 = 20%)		− 0.01 ***		− 0.02 ***		− 0.02 ***
Household composition (two adults, no children)		Reference		Reference		Reference
Single person HH		0.10 ***		0.32 ***		− 0.00
Single parent HH		0.01		1.47 ***		0.92 ***
Two adults, children		− 0.10 ***		1.36 ***		0.88 ***
Other HH, no children		0.10 ***		1.64 ***		1.25 ***
Other, children		0.01		2.66 ***		1.69 ***
Country of birth (Local)		Reference		Reference		Reference
EU		0.14 ***		0.22 ***		0.36 ***
Non-EU		0.10 ***		0.41 ***		0.47 ***
Age of Household head (30–49)		Reference		Reference		Reference
18–29		− 0.06 *		0.42 ***		0.24 ***
50–64		− 0.04 **		− 0.23 ***		− 0.17 ***
65–80		− 0.17 ***		− 0.49 ***		− 0.52 ***
Number of working adults in HH (2)		Reference		Reference		Reference
0		0.11 ***		− 0.10 ***		0.19 ***
1		0.07 ***		0.04 *		0.16 ***
3 +		− 0.07 *		0.37 ***		0.18 ***
Income Quintile (1)		Reference		Reference		Reference
Income Quintile 2		− 0.40 ***		− 0.76 ***		− 0.84 ***
Income Quintile 3		− 0.65 ***		− 1.13 ***		− 1.24 ***
Income Quintile 4		− 0.87 ***		− 1.56 ***		− 1.76 ***
Income Quintile 5		− 1.11 ***		− 2.11 ***		− 2.38 ***
N	27	27	27	27	27	27
Observations	224,723	224,723	224,717	224,717	224,717	224,717
Marginal R2/Conditional R2	0.015/0.081	0.111/0.179	0.053/0.377	0.271/0.559	0.064/0.320	0.243/0.486

^*^p < 0.05 **p < 0.01 ***p < 0.001

**Table 5:** Country-level variables from multi-level model—housing conditions

	M3	M4	M5	M6	M7
GDP per capita (log)	− 0.37*	− 0.40	− 0.34	− 0.18	0.09
Total Residential Loans (as % of GDP)		0.00			
% Owner paying mortgage			− 0.00		
% AROPBHC				0.05**	
Average material deprivation (0–9 index)					0.70*
AIC	203,165	203,167	203,167	203,160	203,162
BIC	203,516	203,528	203,528	203,522	203,524

*p < 0.05 **p < 0.01 ***p < 0.001

See Model 2 for list of household-level predictors

Beginning with Table 3, we start with a null model which simply includes a random intercept that captures country-level differences. Model 1 introduces tenure information, while Model 2 (“Tenure + ”) adds all other socio-economic variables—i.e., the micro-level coefficients contained in Table 4. Models 3–7 add country-level aggregate variables in separate models: GDP per capita, total residential loans as a percentage of GDP, the incidence of mortgaged homeownership amongst all households, the percentage of the population in each country in relative poverty and the country-level average material deprivation score (these country-level coefficients are presented in Tables 5, 6 and 7).

If we first inspect the null model in Table 3, containing only the random intercept, we see that 27% of the variation in the risk of overcrowding and 21% for the severe housing deprivation measure is due to country differences, compared to only 6% for conditions deprivation (as captured by the Intraclass Correlation Coefficient, or ICC (Table 3, Null model).^1^ We take Model 2 (“Tenure + ”) as our reference model. This model contains all the household-level variables and a random intercept for each country. Variation in overcrowding remains much more dependent on country-level differences than does conditions deprivation: country-level variation, as captured by the ICC, for overcrowding represents 29 per cent of total variation in Model 2 for overcrowding and a quarter for the SHD measure, compared to less than a tenth for conditions. Another way of presenting the importance of country-level variation is in terms of the Median Odds Ratio. This captures the odds of deprivation for respondents with the same values on the independent variables from two randomly-selected countries, with the median value taken for all possible country comparisons. In intuitive terms, higher values indicate the greater importance of level-2 (country-level) differences. The median odds ratio in M2 is above 7 for the overcrowding measure and above 4 for the Severe Housing Deprivation measure while it is roughly 1.3 for housing conditions, again supporting the idea that country differences are more important for overcrowding and severe housing deprivation than they are for the deprivation of conditions.

Table 4 presents the full micro-level models, which contain tenure only (M1) and tenure and a wide range of socio-economic characteristics (M2), in addition to a country-level random intercept for each of the three outcome variables of interest. Comparing the coefficients for tenure in these two models, we see that owners, both with a mortgage and without, experience lower rates of all three measures than market renters, and this lower risk remains after controlling for compositional differences (i.e. comparison of effects between M1 and M2). In contrast, in the unadjusted model (M1), reduced-rate renters experience an enhanced risk of all three forms of deprivation relative to market rate tenants, but these effects are fully explained by compositional differences.

Examining the full range of coefficients in Model 2, which contains the random intercept and household-level variables only, we find that some of the variables perform similarly—e.g. tenure, income quintile, housing cost burden—across the three dependent variables. Housing cost burden is of particular interest here since the direction of the effect is the opposite of what was observed in the descriptive analysis. Here, conditional on income and the other socio-economic variables, a higher housing cost burden is associated with *lower* rates of all three outcome variables. The intuition behind this is perhaps that, conditional on income especially, spending more on housing results in fewer housing quality problems. Turning to our summary statistics, the marginal R^2^ captures the proportion of variation explained by the fixed effects (i.e. the within-country coefficients) while the conditional R^2^ captures the proportion of variation explained by both the fixed (within-country) and random (between-country) effects. The model for overcrowding has a greater marginal R^2^ than that for housing conditions, due, in part, to the inclusion of independent variables related to the definition of overcrowding (namely, household composition), and a higher conditional R^2^, due to the greater proportion of variation explained by country-level differences, consistent with the descriptive analysis above.

There are, then, other variables which fall in the same direction but with quite different intensities. For example, neighbourhood problems (noise, pollution, crime) are associated with both forms of housing deprivations but are more strongly related to conditions deprivation than to overcrowding. Non-nationals and larger households experience pronounced rates of both forms of deprivation but face a particularly high risk of overcrowding, as we experienced in the descriptive analysis also.

In terms of differences, conditions deprivation is associated more with houses than apartments, which is the opposite pattern to overcrowding, again reflecting the pattern that we observed in the descriptive analysis. Overcrowding is more strongly associated with household composition than is conditions (which is partly a matter of construction). Age of the household head is more strongly associated with reduced rates of overcrowding. Thus, there are a reasonable number of differences in terms of the socio-economic patterning of conditions and overcrowding deprivation—again, broadly in line with the descriptive analysis conducted earlier.

Tables 5, 6, 7 present country-level coefficients from a series of models. Starting with our models for the deprivation of housing conditions (Table 5), we see that conditions deprivation is negatively associated with GDP per capita (M3). Given the strength of this relationship and the descriptive findings presented above, we retain GDP per capita in the subsequent models (M4 to M7), testing the remaining coefficients while controlling for levels of wealth. There is no relationship between conditions deprivation and Total Residential Loans or the share of mortgaged homeownership (M4 and M5), our proxies for financialisation. Our two poverty measures—material deprivation and, especially, relative income poverty *are* significantly associated with conditions deprivation (M6 and M7). Importantly, once these have been included, much of the effect of GDP per capita has been explained (and it is no longer statistically significant). Thus, poverty rates seem particularly important in explaining between-country differences in conditions deprivation.

**Table 6:** Country-level variables from multi-level model—overcrowding

	M3	M4	M5	M6	M7
GDP per capita (log)	− 1.44***	− 1.13*	− 1.13	− 1.54***	− 1.12
Total Residential Loans (as % of GDP)		− 0.01			
% Owner paying mortgage			− 0.02		
% AROPBHC				− 0.03	
Average material deprivation (0–9 index)					0.49
AIC	132,553	132,555	132,555	132,555	132,555
BIC	132,904	132,916	132,916	132,917	132,916

*p < 0.05 **p < 0.01 ***p < 0.001

See Model 2 for list of household-level predictors

**Table 7:** Country-level variables from multi-level model—EU severe housing deprivation measure

	M3	M4	M5	M6	M7
GDP per capita (log)	− 1.46 ***	− 1.18 **	− 1.15 *	− 1.41 ***	− 1.12 *
Total Residential Loans (as % of GDP)		− 0.01			
% Owner paying mortgage			− 0.02		
% AROPBHC				0.01	
Average material deprivation (0–9 index)					0.50
AIC	57,714	57,715	57,716	57,716	57,716
BIC	58,065	58,076	58,077	58,077	58,077

*p < 0.05 **p < 0.01 ***p < 0.001

See Model 2 for list of household-level predictors

In Table 6, we present the results for an equivalent set of models, this time with overcrowding as the outcome variable. The results here are different. There is, again, a negative association with GDP per capita, but this is now four times stronger (M3). There is now a weak relationship between our proxies for financialisation (Total Residential Loans/GDP and the rate of mortgaged homeownership) and overcrowding, but it runs counter to our expectations—namely, higher rates of these variables are associated with lower rates of overcrowding. Including these variables and the poverty variables (M3-M7) does not result in a substantial moderation of the effect of GDP per capita. Thus, overcrowding does appear to be explained to a greater extent by differences in wealth (GDP per capita).

Finally, in Table 7 we present results from an equivalent model for the combined measure of severe housing deprivation. Here we again see a strong relationship with average wealth (GDP per capita). The housing variables are also more similar to those observed in the overcrowding model (both weak negative relationships). The coefficient for relative income poverty is something of a mid-point between that for the two models of the components while it is very similar to the overcrowding model in respect of material deprivation (M6 and M7). This analysis serves to confirm that, controlling for compositional differences between countries, the two components of severe housing deprivation respond to very different determinants at the country level.

## Discussion

Indicators of housing quality problems in Europe are typically analysed for one of two purposes: either to compare the incidence of such problems in the different nations of Europe (and sometimes for different groups within these nations) or to evaluate competing theories in terms of the extent to which they account for housing outcomes. There is, however, limited agreement about how housing deprivation should be understood or measured. The literature shows, at a general level, that housing quality problems are not highly correlated, indicating that different types of housing quality problems affect different households, and that, in relation to the EU’s measure of severe housing deprivation, that the overcrowding measure behaves rather differently from the three housing deprivations they also monitor (i.e. having a dwelling that is too dark, that has a leak or a damp or mould problem, or that lacks indoor bath/shower or toilet).

Whereas previous contributions have either examined the measurement properties of these indicators or explored their relationship with country-level predictors separately, in this paper we have sought to bring these two important avenues of enquiry together—that is, to shed light on these measures and in so doing help to advance understanding about the measurement of (severe) housing deprivation as well as theoretical debates about the determinants of housing standards. In doing so, we believe that our research advances the state-of-the-art in this field.

Nonetheless, an important weakness of our study is that, given the restricted degrees of freedom in our country-level analysis, we have limited capacity to parse between competing causal explanations at the macro-level. Thus, while we stress the significance of levels of wealth in explaining overcrowding and severe housing deprivation, Borg and Guio (2021: 214) observe a macro-level relationship between severe housing deprivation and the proportion of households who own their homes outright. They suggest that outright ownership can be interpreted as ‘a crude indicator of historical and institutional factors that affect the availability and quality of housing in eastern and southern regimes’. This is a somewhat ambiguous interpretation and we do not find this to be an intuitive reading of the observed empirical relationships given that, within countries, outright ownership is associated with *lower* levels of housing deprivation, which cuts against the idea that this is the underlying determinant of severe housing deprivation rates at the country-level. But we must accept our ability to parse the explanations empirically is limited. This is an issue given that the housing systems where outright ownership is dominant tend to be located in Central and Eastern Europe in particular, where levels of economic development are lower (see also Hick et al., 2022). To examine this further, we re-run our final multi-level moscdel as per Table 7 above, with the proportion of outright owners and GDP per capita as macro-level explanatory variables. We find that the proportion of outright owners is statistically significant only before we add GDP per capita into the statistical model, while GDP remains a significant predictor even after we include this measure of tenure balance into the model (results not presented here, but available from the authors on request).

## Conclusion

In this paper we have presented a joint analysis of the measurement and determinants of severe housing deprivation and its two component measures, drawing on data from the EU Statistics on Income and Living Conditions survey for 2016 for 27 European nations. Our results have relevance for measurement, theory and policy and can be summarised as follows. First, we have shown the importance of the aggregation rules for any composite housing deprivation measure. If we classify as deprived households that experience *any* deprivation (any of the housing deprivation *or* overcrowding items) then deprivation levels exceed 50% in some countries. Counting severe housing deprivation as experiencing *both* one of the housing deprivations *and* overcrowding on the other hand results in levels of severe housing deprivation that are extremely low in the richer nations. The weak relationship between the deprivation in housing conditions and overcrowding results in very low severe housing deprivation rates in some—mostly richer—countries and suggests a level of severe housing deprivation that is substantially lower than for either of the measures of conditions or overcrowding alone.

Overlap measures such as the severe housing deprivation indicator can be justified if there is a strong conceptual case for only considering the absence of adequate housing conditions (that is, an indoor toilet and bath or shower, adequate daylight or the presence of a leaking roof or damp ceilings) *if* the household is also overcrowded. Or, conversely, that overcrowding matters if and only if there is also the presence of one of these housing deprivation problems. But such an account appears to be lacking (for a related discussion of overlap measures of poverty, see also Hick, 2014). The consequence is that countries and/or groups with very low levels of severe housing deprivation can exhibit non-trivial rates of one of the two forms of deprivation, which can give us a misleading sense of the scale of housing conditions problems. To take just one example, nearly one in five (19%) of households headed by someone aged 50–64 experienced conditions deprivation, but just 3% of this group experienced severe housing deprivation. Thus, reliance on an intersection measure can substantially alter our understanding of the extent of housing deprivation problems. Absent a compelling reason for only focussing on the intersection, and given their weak empirical relationship, there would appear to be a case for considering these items separately.

Second, and relating to the estimates produced by these measures, we show in descriptive analysis that the patterning of conditions deprivation and overcrowding is reasonably similar, with many of the same groups being at risk. Dwelling type and area variables were notable exceptions, with conditions deprivation more common amongst households living in detached properties and in rural areas while overcrowding was more common in rental properties and in urban ones. This gives some cause for concern given that these variables themselves are important in relation to housing. Third, findings from both our descriptive as well as our regression-based analyses demonstrate that tenants (both market-rate and those paying rent at a reduced rate) have substantially higher rates of housing deprivation than owners.

Fourth, and of relevance for theory, the macro component of our multi-level models show that, at the country-level and controlling for compositional differences between countries, the two component indicators of the severe housing deprivation measure respond to quite different determinants. We find that there is a significant relationship between GDP per capita, and a weak relationship between material deprivation, and overcrowding and the combined measure (but no relationship between these measures and relative income poverty) and significant relationship between both material deprivation and relative income poverty (but not GDP per capita) and conditions deprivation. This analysis endorses the significance of economic variables for predicting country-level housing deprivation rates, though as we see the component measures of severe housing deprivation vary by different economic variables.

We have explored whether housing system financialisation, proxied by our Total Residential Loans to GDP measure as well as the proportion of the population that are mortgaged homeowners, is associated with our three measures of housing deprivation at the country level. We find no relationship between these variables and conditions deprivation and a weak *negative* (albeit non-significant) relationship between them and overcrowding as well as the combined severe housing deprivation measure, contrary to our expectations. Differences in housing matter within countries (e.g. the elevated rates of housing deprivation for renters vis-à-vis owners) but system differences between countries appear to matter much less. What is shown is that wider economic circumstances are important determinants of differences in housing deprivation between countries.

In terms of policy, given that severe housing deprivation captures the experience of both deprivation in relation to housing conditions *and* overcrowding, reducing rates can be achieved by targeting either component. Overcrowding rates have reached a much lower bound in some countries than have conditions deprivation, which suggests that overcrowding is more amenable to reduction. Our analysis consistently finds that wealth is strongly associated with overcrowding, which indicates that further improvements to GDP per capita would be likely to reduce overcrowding rates further in poorer Member States. Reducing housing conditions problems seems harder, and no nation has been hugely successful at it. However, this appears much more closely associated with country-level poverty rates, which suggests that redistributive programmes that raise the income floor may enable families to address problems with housing conditions.

## Data Availability

This paper is based on an analysis of EU Statistics on Income and Living Conditions microdata 2004-2019, release 2 in 2020. Information about this data release can be found at https://ec.europa.eu/eurostat/documents/203647/203704/EU+SILC+DOI+2020v2.pdf. These data are held by Eurostat. Data are made available for scientific purposes only to researchers working at a recognised research entity. To apply for access to the microdata, please see the following file: https://ec.europa.eu/eurostat/documents/203647/771732/How_to_apply_for_microdata_access.pdf.

## Footnotes

1 As we add more explanatory variables to a logistic regression model we introduce differences in the underlying scale so subsequent models are not strictly comparable, but the significant discrepancy in terms of the proportion of variation explained at the country-level for these three variables is confirmed in these models.
